# Implications of Non-compliance in the Management of Chronic Polycystic Ovarian Syndrome

**DOI:** 10.7759/cureus.79501

**Published:** 2025-02-23

**Authors:** Avarie Willette, Taylor Nicely, Elliot Cazes

**Affiliations:** 1 Obstetrics and Gynecology, Lake Erie College of Osteopathic Medicine, Tampa, USA; 2 School of Medicine, Lake Erie College of Osteopathic Medicine, Bradenton, USA; 3 Obstetrics and Gynecology, New Tampa OB/GYN, Tampa, USA

**Keywords:** gynecology, hirsutism, irregular bleeding, patient education, polycystic ovary syndrome (pcos)

## Abstract

Polycystic ovarian syndrome (PCOS) is a reproductive endocrine disorder characterized by an imbalanced luteinizing hormone (LH) to follicle-stimulating hormone (FSH) ratio, which can lead to a range of complications, including infertility, metabolic disturbances, and cardiovascular disease. Diagnosis is based on the Rotterdam criteria, which require the presence of at least two of three features: oligo- or anovulation, clinical and/or biochemical hyperandrogenism, and polycystic ovaries. This case study follows a patient, from ages 28 to 34, who presents with complications while managing her PCOS. Diagnostic tests first confirmed excess androgen production and oligo-amenorrhea, leading to the initiation of metformin hydrochloride (HCl) and Lo Loestrin Fe, a low-estrogen oral contraceptive. However, the patient failed to comply with her medication regimen. Four years later, the patient returned with ongoing irregular bleeding episodes and severe hirsutism. This case addresses the importance of understanding medication non-compliance in the management of chronic conditions such as PCOS, where factors such as financial hardship and lack of understanding about the condition can hinder treatment adherence. Improved patient education, proactive management of medication side effects, and assistance with healthcare costs are essential to improving adherence and long-term health outcomes.

## Introduction

Polycystic ovarian syndrome (PCOS) is a reproductive endocrine disorder affecting 5-20% of women worldwide [1]. Complications resulting from PCOS can be life-altering and life-threatening if left untreated, including diabetes and cardiovascular disease. The revised Rotterdam criteria are used by physicians as a guideline for diagnosing PCOS. It states that patients must have two out of the following three criteria for diagnosis: oligo- and/or anovulation; clinical and/or biochemical signs of hyperandrogenism; and polycystic ovaries. Other etiologies such as congenital adrenal hyperplasias, androgen‐secreting tumors, and Cushing’s syndrome must be ruled out before a PCOS diagnosis can be made [2].

Clinical manifestations

It can be difficult to diagnose PCOS due to differing clinical manifestations throughout the population. Many women are first diagnosed with PCOS after seeking fertility treatment due to difficulties conceiving. This is a direct consequence of oligo- and/or anovulation. Without treatment, it may be extremely difficult to nearly impossible for these women to conceive. Even with treatment, miscarriage rates are increased in women with PCOS, 71.4% versus 53.6% in the general population [3].

Additionally, some women initially seek treatment for PCOS due to the effects of hyperandrogenism. The underlying mechanisms by which PCOS causes hyperandrogenism are multifactorial, involving a combination of hormonal imbalances and disrupted ovarian function. Excess androgens in the female body can produce excessive acne, male-patterned hair loss, also known as androgenic alopecia, and excess hair growth. Hirsutism is typically found on the face, arms, and chest of women with untreated hyperandrogenism.

Polycystic ovaries are both the hallmark and a key manifestation of the condition. In order to classify as having polycystic ovaries, a patient must have 12 or more follicles in each ovary measuring 2-9 mm [4]. However, numerous studies have found the number of follicles in those diagnosed near the upper end of 20, and so many call for a higher threshold for diagnosis [5]. Despite this, the Rotterdam criteria of greater than 12 remain the most widely used diagnostic tool for PCOS.

The metabolic effects of PCOS are closely studied because they can contribute to severe, life-threatening complications. Insulin resistance presents in upward of 50% of patients with PCOS [6]. Because of this high rate of insulin resistance, it is recommended that those who are obese and diagnosed with PCOS should be tested for metabolic disorders. While the development of cardiovascular disease is multifactorial, obesity and hypercholesterolemia play a large part, studies have found a link between PCOS and heart disease independent of BMI [7]. Given that heart disease is the leading cause of death in the United States, it is crucial to effectively manage conditions like PCOS in order to reduce mortality rates among women.

## Case presentation

The patient presented to obstetrics and gynecology (OB-GYN) in 2018 at the age of 28 for her annual examination. At the time, the patient complains of irregular cycles with her last menstrual period one year before her presentation to the office. The patient experienced menarche at the age of 17 and has had irregular menstrual cycles since the onset of menses. The patient was diagnosed with PCOS by her primary care provider but was not on any medications at that time. Upon physical examination, moderate to severe hirsutism was noted on the arms, trunk, and breasts. Hormone panels, including follicle-stimulating hormone (FSH), luteinizing hormone (LH), estradiol, progesterone, and testosterone, were ordered. The patient was instructed to follow up to discuss laboratory results once obtained. 

Laboratory results are listed in Table 1. The patient had satisfied at least two of the three criteria for diagnosis of PCOS at this time, including excess androgen production and oligo-amenorrhea. The patient’s testosterone level was 103.20 ng/dL with a reference range for a female from 2.9 to 48.1 ng/dL. The patient's LH-to-FSH ratio was over 2.84. Laboratory values were discussed with the patient one month after obtaining values. It was decided to start on metformin hydrochloride (HCl) and Lo Loestrin Fe to regulate her menstrual cycles and alleviate laboratory abnormalities contributing to her irregular cycles and hirsutism. Instructions were to follow up to discuss the effectiveness of treatment. 

**Table 1:** Hormone panel obtained in 2018 FSH: follicle-stimulating hormone; LH: luteinizing hormone

Parameters	Patient values	Reference range
LH	20.2 mIU/mL	Follicular phase: 2.4-12.6 mIU/mL; ovulation phase: 14.0-95.6 mIU/mL; luteal phase: 1.0-11.4 mIU/mL; postmenopause: 7.7-58.5 mIU/mL
FSH	7.1 mIU/mL	Follicular phase: 3.5-12.5 mIU/mL; ovulation phase: 4.7-21.5 mIU/mL; luteal phase: 1.7-7.7 mIU/mL; postmenopause: 25.8-134 mIU/mL
Estradiol	55.3 pg/mL	Follicular phase: 12.4-233 pg/mL; ovulation phase: 41.0-398 pg/mL; luteal phase: 22.3-341 pg/mL; postmenopause: <5-138 pg/mL
Progesterone	0.470 ng/mL	Follicular phase: 0.057-0.893 ng/mL; ovulation phase: 0.121-12.0 ng/mL; luteal phase: 1.83-23.9 ng/mL; postmenopause: 0.03-0.126 ng/mL
Testosterone	103.20 ng/dL	2.9-48.1 ng/dL

The patient returned to the OB-GYN office the following year, 2020, for her annual examination and stated she had not been compliant with her medications. She declined a hormone panel due to a lack of insurance. The patient was instructed to follow up yearly for her annual examination or as frequently as needed.

Four years later, the patient is now a 34-year-old female who presents to the OB-GYN complaining of irregular bleeding episodes lasting six weeks. The patient reports experiencing episodic bleeding lasting several days, followed by a complete cessation before reoccurrence days later. Severe hirsutism was found at this time on the patient's trunk and arms. The patient was educated that irregular bleeding episodes were likely due to her PCOS. The patient was instructed that medication adherence could resolve her irregular menstrual cycles. Laboratory values were obtained to assess the patient's current hormone levels and establish insight into the progress of her condition. Results are listed in Table 2. The LH-to-FSH ratio was 1.95. Testosterone level was not obtained as the lab reported the specimen integrity was compromised. A transvaginal ultrasound was ordered. The patient was instructed to adhere to the medication regime and follow up for repeat laboratory values. 

**Table 2:** Hormone panel obtained in 2024 FSH: follicle-stimulating hormone; LH: luteinizing hormone

Parameters	Patient values	Reference range
LH	16.0 mIU/mL	Follicular phase: 2.4-12.6 mIU/mL; ovulation phase: 14.0-95.6 mIU/mL; luteal phase: 1.0-11.4 mIU/mL; postmenopause: 7.7-58.5 mIU/mL
FSH	8.2 mIU/mL	Follicular phase: 3.5-12.5 mIU/mL; ovulation phase: 4.7-21.5 mIU/mL; luteal phase: 1.7-7.7 mIU/mL; postmenopause: 25.8-134 mIU/mL
Estradiol	101 pg/mL	Follicular phase: 12.4-233 pg/mL; ovulation phase: 41.0-398 pg/mL; luteal phase: 22.3-341 pg/mL; postmenopause: <5-138 pg/mL
Progesterone	0.7 ng/mL	Follicular phase: 0.057-0.893 ng/mL; ovulation phase: 0.121-12.0 ng/mL; luteal phase: 1.83-23.9 ng/mL; postmenopause: 0.03-0.126 ng/mL

## Discussion

The patient consistently demonstrated abnormal laboratory values and struggled to manage her polycystic ovarian syndrome (PCOS), leading to suboptimal health outcomes. These outcomes were ultimately due to non-compliance and loss of follow-up multiple times during her care. Healthcare professionals need to investigate why the patients may not be compliant with their medications to gain insight into any factors that influence their decisions. In this case, a potential factor contributing to non-compliance could be a potential financial burden related to lack of insurance. Lack of insurance coverage of medication cost was reported as the primary reason for medication non-compliance in 20.2% of individuals surveyed by the Medicare Current Beneficiary Survey (MCBS) [8]. 

Medication side effects could have also contributed to her decision to not follow her regimen. Metformin, one of the patient’s prescribed medications, has many potential side effects, including GI disturbance and impaired kidney functioning. Metformin was taken off the market after concerns for lactic acidosis but was reintroduced in 1995 after it was deemed safe [9]. While Metformin is safe and proven effective in treating the metabolic effects of PCOS, the potential for serious side effects could still deter patients from using this medication.

A factor discussed by the patient with her healthcare provider was the loss of healthcare coverage; therefore, medication costs may have been a driving factor in non-compliance. In a study completed by the Medicare Current Beneficiary Survey, the most commonly cited reason for failure to fill medications was the fear of cost in 55.5% of those surveyed [8]. Had this been the reason for non-compliance in this patient, resources for assistance could have been provided. Ultimately, a deeper dive into why the patient was non-compliant could have produced an opportunity to intervene, and this could have changed health outcomes.

Non-compliance among patients, particularly those with PCOS, is a significant challenge that impacts health outcomes. Li et al. studied 90 women who were given treatment plans for their PCOS and measured levels of adherence using interviews. They found only 23 of the 90 women were completely adherent to their treatment. This was only 25.5% of those studied. Reasons for non-compliance included lack of convenience and concerns about adverse drug reactions [10]. Establishing reasons for non-compliance could guide healthcare professionals in addressing these issues, such as providing more patient education on the side effect profiles of all medications prescribed. This intervention could encourage women to be engaged in their treatment and lead to positive effects on their health.

Patient compliance to treatment is not only crucial to the individual patient's health but is also needed to further examine the efficacy of treatment options. Without full compliance, it is extremely difficult to determine the best course of action for treatment. Parker et al. describe in their systematic review of treatment adherence in PCOS that adherence rates vary greatly from one study to the next. The ranges included in the review were from 21.7% to 86% of patient adherence. Parker et al. cautioned healthcare professionals that patient compliance significantly influences clinical outcomes, particularly in PCOS cases [11]. 

One limitation of this case is the difficulty in applying a one-size-fits-all solution to non-compliance, as it is a highly individualized issue that often requires tailored strategies. However, a key takeaway is that effective management of chronic conditions goes beyond prescribing the correct treatment regimen; it also requires ensuring that patients are adequately educated about their condition and the importance of their medication regimen. Ultimately, even with the best treatment plan, a patient’s willingness and active participation are crucial for their improvement.

## Conclusions

The patient initially presented to the OB-GYN in 2018 with complaints of irregular menstrual cycles. After laboratory results were obtained, a diagnosis of PCOS was confirmed due to satisfaction of at least two of the three Rotterdam criteria. Metformin HCl and Lo Loestrin Fe were prescribed to address her symptoms.

However, when the patient returned two years later, she admitted to non-compliance with her prescribed medications, and she declined a hormone panel due to financial difficulties. Four years later, the patient returns and again reports irregular bleeding episodes, highlighting the ongoing challenges in managing her condition.

This case demonstrates the importance of addressing the barriers to healthcare access and medication adherence. It is essential for healthcare providers to consider social and financial factors that may contribute to non-compliance. Addressing medication non-compliance requires providers to enhance patient education, guide side effect management, clarify treatment timelines, and offer financial assistance when needed. By taking a comprehensive and multifaceted approach, physicians can improve adherence and patient outcomes.
